# Γ*δ* T Cell-Based Immunotherapy in Melanoma: State of the Art

**DOI:** 10.1155/2019/9014607

**Published:** 2019-05-23

**Authors:** F. Toia, A. B. Di Stefano, S. Meraviglia, E. Lo Presti, R. Pirrello, G. Rinaldi, F. Fulfaro, F. Dieli, A. Cordova

**Affiliations:** ^1^Department of Surgical, Oncological and Oral Sciences, Plastic and Reconstructive Surgery Section, University of Palermo, 90127, Italy; ^2^Department of Biopathology, Central Laboratory of Advanced Diagnosis and Biomedical Research (CLADIBIOR), University of Palermo, 90127, Italy; ^3^Department of Surgical, Oncological and Oral Sciences, Oncology Section, University of Palermo, 90127, Italy

## Abstract

Metastatic melanoma is still associated with a poor prognosis, and there is increasing interest in immunotherapy alone or in combination with other adjuvant therapies. Γ*δ* T lymphocytes play a pivot role in the immune response against cancer, but while *γδ*-based immunotherapy is already a clinical reality for several solid tumors, data on melanoma are still limited and fragmented. This systematic review presents preclinical and clinical evidence for a role of *γδ* T lymphocytes in immunotherapeutic strategies for advanced melanoma and discusses research state of the art and future perspectives. Current strategies focus on in vivo stimulation, and ex vivo adoptive therapy and vaccination; results are promising, but further studies are needed to better investigate the interactions in tumoral microenvironment and to improve clinical efficacy of immunotherapeutic protocols.

## 1. Introduction

Metastatic melanoma is still associated with a poor prognosis. Current therapeutic protocols are often ineffective: chemotherapy shows a low efficacy (15%), short duration of response (6 months), and no significant improvement in overall survival; BRAF-targeted therapies are highly effective in metastatic BRAF mutated melanoma (about 60%), but show a short-lived response, while immunotherapy shows a low frequency but extremely durable tumor response [1–3].

In the last years, there is increasing interest in immunotherapy alone or in the combination with targeted therapy [2]. Gamma delta T (*γδ*) lymphocytes are of particular interest due to their potent antitumoral effect via cytotoxicity and relative ease of culture in vitro. Promising reports from clinical trials support their use as immunotherapeutic agents, either via adoptive transfer of ex vivo expanded *γδ* T cells or in vivo activation with aminobisphosphonates, but data concerning melanoma are still limited [4–7].

This systematic review presents preclinical and clinical evidence for a role of *γδ* T lymphocytes in immunotherapeutic strategies for advanced melanoma and discusses research state of the art and future perspectives.

## 2. Materials and Methods

A systematic literature search was conducted in the PubMed database for articles published between November 01, 2008, and October 31, 2018. The following key words were used: “(melanoma[Title/Abstract]) AND (Vgamma9Vdelta2[Title/Abstract] OR gammadelta[Title/Abstract] OR gamma delta OR *γδ* [Title/Abstract]) and immunotherapy[Title/Abstract]”. Article selection was performed according to the following criteria for inclusion and exclusion:

- inclusion criteria: preclinical or clinical research papers concerning the potential immunotherapeutic role of *γδ* T lymphocytes in advanced melanoma;
- exclusion criteria: papers in language other than English, reviews.

Two reviewers independently screened all search results, abstracts, and full texts. Further search included relevant references from selected articles. Data on type of paper, number of patients or research animals, laboratory tests, and results were extrapolated from selected articles.

Data were analyzed to summarize current evidence on the following questions:

- What is the potential role for a *γδ*-based immunotherapy in advanced melanoma?
- What are the possible targets and strategies for a *γδ*-based immunotherapy in advanced melanoma?
- Is there any difference in possible immunotherapeutic approach for BRAF mutated melanoma?

## 3. Results and Discussion

**Figure 1:**
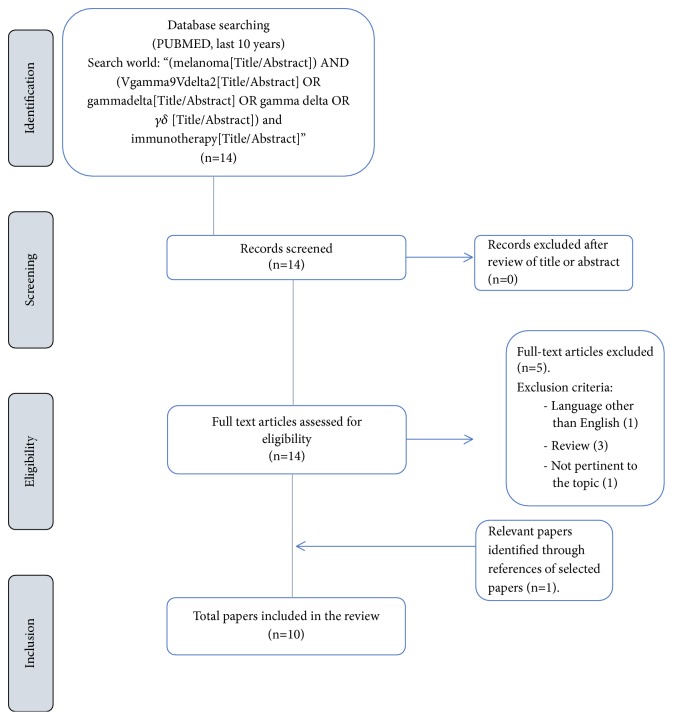
Flow chart of search and selection strategy.

Initial search retrieved 14 articles. Nine articles fulfilled inclusion and were selected based on full text review. One further relevant article was identified and a total of 10 papers were included in the study.  Figure 1 presents a detailed flow diagram of our literature search strategy and results. Studies' design and results are summarized in Table 1.

**Figure 2:**
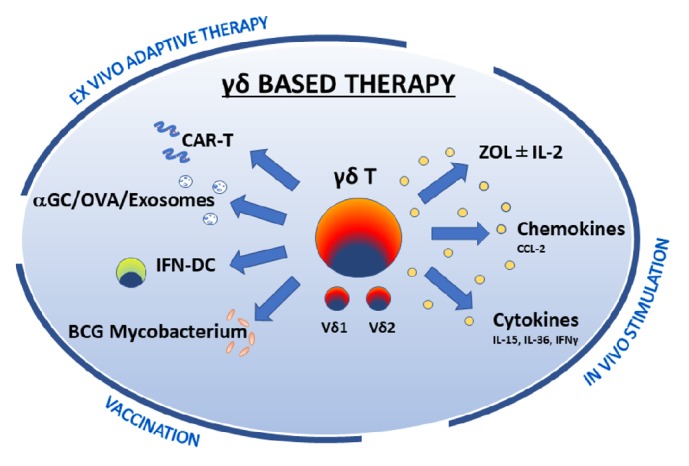
Schematic representation of the main preclinical and clinical research lines on *γδ* T cell-based immunotherapy in melanoma.

Literature review revealed that strong preclinical and limited clinical evidence supports the role of *γδ* T cells in immunotherapeutic strategies for advanced melanoma. Clinical data on *γδ*–based immunotherapy in melanoma are limited, with only 3 clinical studies on a total of 24 patients evaluating different immunotherapeutic protocols for advanced melanoma [8–10] and most studies being performed on melanoma cell lines. Investigated strategies include in vivo stimulation, or an ex vivo adoptive therapy or vaccination. Some authors propose direct stimulation through nitrogen-containing bisphosphonate ± IL-2, IL-36, IL-15, or intralesional-BCG, while others proposed a vaccination strategy through dendritic cells or dendritic cell–derived exosomes; adoptive cell therapeutic approach includes RNA-transfection of *γδ* T cells with a chimeric antigen receptor or an *αβ* T cell receptor (Figure 2).

Future prospective on the development of *γδ*-based immunotherapy for melanoma will be discussed on the basis of selected studies and other relevant literature—mainly descriptive—on *γδ* T cells and melanoma.

V*δ*1 and V*δ*2 T cells are the main represented subpopulations of *γδ* T cells, which account for up to 10% of circulating lymphocytes in adult and healthy humans. V*δ*2 T cells are best represented in the peripheral blood and lymphoid organs, V*δ*1 T cells, instead of mucosal and epithelial tissues. V*δ*2 T cells recognize metabolites of host mevalonate and microbial nonmevalonate pathway (phosphoantigens) [11], opposite to V*δ*1 T cells which have some candidate antigens of stressed and tumor cells, a different immune response bound to different pathogens. Furthermore, all *γδ* T cells share characteristics of both adaptive and innate immunity, posing tricking questions still debated by immunologists about their belonging to one branch or the other of immunity. Nevertheless, new discoveries are regularly published which describe features of *γδ* T cells belonging to both branches of immune system [12, 13]. Notably *γδ* T cells have also the capacity to effectively kill tumor cells [14] with granzyme and perforin, and there are some hints about phagocytosis too [15]. Furthermore, they are activated in a MHC-unrestricted way, whose expression is typically lost in cancer cells that try to escape to immune-surveillance. Then, their killing ability with the freedom from the MHC system makes *γδ* T cells a very interesting candidate for immunotherapies.

Several studies have shown that *γδ* T cells are an important component of tumor-infiltrating lymphocytes and have a positive prognostic role in patients affected by different types of cancer; a recent analysis of ~18,000 transcriptomes from 39 human tumors identified tumor-infiltrating *γδ* T cells as the most significant favorable cancer-wide prognostic signature [16–18]. In melanomas, *γδ* T cells, expressing either V*γ*9/V*δ*2 or V*δ*1 TCRs, have been found within tumor-infiltrating lymphocytes (TILs) [19–21]. Previous studies from our group showed that *γδ* T cells are the most represented subset of melanoma-infiltrating lymphocytes, displaying an activated phenotype and a strong in vitro cytotoxic activity toward melanoma cells. We showed a correlation among *γδ* TILs and melanoma stage, and an inverse correlation of peripheral *γδ* T with mortality and relapse rates in metastatic melanoma [21, 22]. Wistuba-Hamprecht K. et al. also showed an interesting correlation between V*δ*2+ high frequencies and V*δ*1+ low frequencies with favorable overall survival of melanoma patients treated with ipilimumab [23].

While *γδ*-based immunotherapy is already a clinical reality for other solid tumors, research on *γδ* T cells and melanoma is mostly at a preclinical descriptive stage, and their immunotherapeutic potential has not been deeply investigated. Γ*δ* immunotherapeutic strategies have already proved their efficacy in several preclinical and clinical studies on other solid and nonsolid tumors. Most studies focused on the role of N-BPs (nitrogen-containing bisphosphonates) ± IL-2, which proved to be able to induce immunologic and clinical responses, potentially providing a substantially increased window for more specific/targeted approaches to be administered [4–7].

Several studies in this review focused on the well-known sensitizing role of N-BPs on *γδ* T cells.

Hodgins et al. evaluated the efficacy of zoledronate and alendronate to improve the V*γ*9V*δ*2 T cells effect both in vitro and in vivo. In vitro, both zoledronate and alendronate proved to be able to sensitize melanoma cells to V*γ*9V*δ*2 T cells, whose subsequent addition determined a significant and dose-dependent reduction in tumor cell viability; liposomal alendronate was 5 times less potent than liposomal zoledronate in vitro and caused less toxic side effects in an in vivo mice model. In vivo, combined injection of alendronate and *γδ* T cells delayed tumor growth in an experimental metastatic lung mouse model; tumor cells viability correlated with IFN-*γ* concentration, suggesting that cell kill was due to the activation of *γδ* T cells. They also showed that free N-BPs can sensitize cancer cells more efficiently than their liposomal formulations, which on the other hand show an enhanced passive accumulation and retention within solid tumors [24].

Nieda et al. focused on *γδ* stimulation with zoledronate and IL-2, mediated by particular dendritic cells, differentiated from CD14 monocytes in the presence of interferon-*α* (IFN *α*) and granulocyte/macrophage-colony stimulating factor (GM-CSF), known as IFN-DCs (interferon dendritic cells). They showed that IFN-DCs exhibited a stronger capacity to stimulate autologous CD56+ V*γ*9*γδ*T cells highly producing IFN*γ* in the presence of zoledronate and interleukin (IL)-2. Also, this stimulation increased the number of cytotoxic CD8+ T cells through the expansion of CD56+ V*γ*9*γδ*T cells and the authors proposed the CD56 high+ IFN-DCs as hypothetical vaccine in immunotherapies for melanoma patients [25].

Two in vivo studies on stimulation of *γδ* T cells with zoledronate reported limited clinical efficacy and suggested combination strategies for improving clinical outcomes. In a clinical phase II study, 21 patients with solid tumors, of which 6 had malignant melanomas, were selected and treated with a combination of zoledronate and low-dose IL-2. The treatment protocol was generally well tolerated, with mild adverse effects. All patients showed an in vivo expansion in circulating *γδ* T cells after the first cycle. There was a significant increase in the proinflammatory cytokine IFN-*γ* with a positive correlation with expanded *γδ* T cells. Although there were no objective tumor responses (according to the RECIST criteria) within both cohorts of solid tumors, 1 patient with melanoma with prior progressive disease experienced disease stabilization. Also, higher baseline and increasing VEGF serum levels during the first week of treatment with zoledronic acid correlated with poor prognosis. These data indicate a possible role for in vivo *γδ* T cell stimulation, but limited efficacy, and suggest the need for combining immunotherapeutic approaches with anti-VEGF compounds in future clinical trials [26]. Also Nicol et al. pointed out a safe and tolerable profile of zoledronate, but limited clinical efficacy, in their phase I study on metastatic patients with solid primary tumors, of which 7 had melanomas; *γδ* T cells were expanded ex vivo and adoptively transferred in combination with zoledronate administration. Γ*δ* T cells had an activated effector memory phenotype, expressed chemokine receptors predictive of V*γ*9V*δ*2 homing to peripheral tissues, and were cytotoxic in vitro against tumor targets, but most patients progressed despite therapy. Better *γδ* expansion was achieved in patients not pretreated with zoledronate. Authors also observed that the percentage of Tregs in the blood of melanoma patients was significantly higher than controls and correlated with poor *γδ* T cells expansion, and suggested that depletion of Tregs from patient mononuclear cells may be a prerequisite for successful *γδ*-based adoptive therapy [9]. Of note, further evidence for a clinical role of zoledronate for in vivo stimulation in melanoma patients came from Laggner et al., who reported a case of regression of pulmonary and bony metastases in a patient with malignant melanoma following palliative treatment with systemic zoledronate and localized radiotherapy to the bone [27].

Other authors tested a different *γδ* T cells stimulation strategy which relies on injection of* Mycobacterium bovis* bacille Calmette-Guérin (BCG). In a study on 8 patients with stage III in-transit melanoma, epidermic injection of BCG into metastatic melanoma sites showed a 90% regression of injected tumors and 17% regression of uninjected tumors, likely through V*γ*9V*δ*2 T cells recruitment on melanoma sites. However, even this approach did not produce durable responses due to the transient nature of the BCG-induced inflammation [8].

The complex and intricate interactions between tumor cells, tumor microenvironment, and tumor-infiltrating immune cells result in a balance between tumor-promoting and tumor-controlling effects, and *γδ* T cells functions are often diverted or impaired by immunosuppressive signals originating from the tumor microenvironment. Chemotactic signals, as well as chemokines/cytokines, could orchestrate this balance. For this reason, other researchers concentrated on the stimulating role of cytokines on *γδ* T cells. Wang et al. showed that IL-36*γ* effectively promoted IFN-*γ* production by *γδ* T and NK cells; also, tumoral expression of IL-36*γ* greatly inhibited tumor growth and metastasis in an in vivo mouse melanoma model, mainly through IFN-*γ*. Based on their results, the authors proposed IL-36*γ* as immunotherapeutic weapon to boost the efficacy of tumor vaccination [28]. In another preclinical study in a mouse melanoma model, Lança et al. showed a protective role for CCR2/CCL2 through the recruitment of *γδ* T cells. Chemokine CCL2 and its receptor CCR2 are involved in the accumulation of *γδ* TILs, which produce IFN-*γ* and display potent cytotoxic functions. Also, CCL2 directed *γδ* T cell migration toward tumor extracts in vitro, while the lack of *γδ* TILs in TCR*δ*-deficient, but also in CCR2-deficient, mice enhanced tumor growth in vivo. They also demonstrated that human V*δ*1 T cells, but not their V*δ*2 counterparts, express CCR2 and migrate to CCL2, and opened new perspectives on CCL2 as promising target for manipulation of V*δ*1 T cells in cancer immunotherapy [29]. Conlon et al. investigated instead the role of recombinant human (rh) IL15 in a clinical phase I study on 11 patients affected by metastatic melanoma and observed an rhIL15-mediated activation of monocytes, NK, and *γδ* T cells, with no objective remissions in all patients, with the best response being stable disease, reinforcing the idea that combination therapies could be better immunotherapeutic options [10].

Harrer et al. explored a novel approach for adoptive T cell therapy based on mRNA electroporation of melanoma-specific antigen receptors into *γδ* T cells. They proposed a GMP protocol for the expansion and mRNA transfection of *γδ* T cells and gp100/HLA-A2-specific TCR or MCSP (melanoma-associated chondroitin sulfate proteoglycan)-specific chimeric antigen receptor (CAR). Zoledronate-mediated expansion of *γδ* T cells directly from PBMC proved to be more efficient than expanding MACS isolated *γδ* T cells; expanded *γδ* T cells could be efficiently transfected using mRNA electroporation. They also observed that RNA-transfected *γδ* T cells responded to melanoma cells with antigen-specific cytokine secretion, specifically lyse melanoma cells, and retained their intrinsic cytotoxic activity toward melanoma cell lines after electroporation [30].

Other emerging therapeutic agents in cancer immunotherapy are exosomes, which started to be used thanks to their ability to improve innate Th1 immunity and to amplify adaptive immune response. Gehrmann et al. tested the effect of the ligands a-galactosylceramide (aGC) and antigen ovalbumin (OVA) exosomes in a melanoma-bearing mice, demonstrating that antigen-loaded exosomes treatment induces *γδ* T cell activation and proliferation, reduction in tumor growth, and T cell infiltration in vivo, besides inducing OVA-specific CD8 *β* T cell responses and boosting CD4*β* T and B cell responses. They also show that antigen-loaded exosomes are more potent than soluble antigens in inducing adaptive immunity, envisaging a role in future immunotherapeutic approach [31].

Despite increasing interest in the combination of BRAF-targeted therapies and immunotherapy, preclinical and clinical data concerning this approach are still limited, and we did not identify any specific study on BRAF mutated melanoma and *γδ* T cells. In a murine melanoma model, Koya et al. showed that vemurafenib did not significantly alter the expansion, distribution, or tumor accumulation of the adoptively transferred lymphocytes with a genetically modified TCR, while it paradoxically increased their in vivo cytotoxic activity and intratumoral cytokine secretion [32]. Wilmott et al. also reported an increase in CD4(+) and CD8(+) TILs with BRAF inhibitors in metastatic melanomas [33]. These data provide support for conducting trials that combine BRAF inhibitors with immunotherapy in the hope of improving and prolonging clinical responses, although Hooijkaas A et al. suggested instead that vemurafenib may negatively affect the immune activity within the tumor [3]. Therefore, the potential effect of targeted therapy on the tumor microenvironment needs further insight and consideration in the design of clinical trials combining targeted therapy and immunotherapy. Also, interaction between BRAF-targeted therapy and *γδ* T cells immune response should be specifically addressed.

Future research should also focus on tumor microenvironment and immune check points as target for a *γδ* T cell-based immunotherapy and on the role of certain subset of *γδ* T cells, which have been reported to be protumoral such as *γδ* T17 cells, *γδ* Tregs, and V*δ*1 T cells [34].

## 4. Conclusions

Overall, current knowledge strongly suggests that *γδ* T cells should be regarded as prominent tool for enhancing antitumor response in melanoma and points out different possible immunotherapeutic strategies. However, data are still fragmented and limited and focus on in vivo stimulation, and ex vivo adoptive therapy or vaccination. Results of current research are promising, but further studies are needed to better investigate the interactions in tumoral microenvironment and to define immunotherapeutic protocols with improved clinical efficacy.

## Figures and Tables

**Table 1:** Characteristics and main results of selected studies.

Paper	Type of paper	N of patients/animals and study design			Results
		Patients (n)	Animals (n)	Laboratory research	
Harrer D.C. et al. BMC Cancer (2017)	Research	NA	NA	Isolated *γδ* T cells transfected through mRNA electroporation with a gp100/HLA-A2-specific TCR and an MCSP-specific CAR.	Zoledronic acid-mediated expansion of *γδ* T cells directly from PBMC is more efficient than expanding MACS isolated *γδ* T cells RNA-transfected *γδ* T cells responded to melanoma cells with antigen-specific cytokine secretion and tumor cell lysis, and retained their intrinsic cytotoxic activity towards melanoma cells after electroporation

Yang J. et al. Fron. Oncol (2017)	Clinical	n. 8 patients with stage III in-transit melanoma treated with IL-BCG	NA	NA	V*γ*9V*δ*2 T cells play a role in IL-BCG-induced melanoma regressions.

Hodgins N.O. et al, Journal of Controlled Release (2016)	Research	NA	NSG mice L-ZOL (toxicity assessment) L-ALD + *γδ* T cells (efficacy assessment)	*In vitro* cell lines A375Ppuro	(i) In vitro, zoledronate and alendronate + Vg9Vd2 T-cells determined a significant and dose-dependent reduction in tumour cell viability. (ii) In vivo, combined injection of alendronate and *γδ* T cells delayed tumour growth in an experimental metastatic lung mouse model

Wang X. et al. Cancer Cell. (2015)	Research	NA	C57BL/6j (B6; H2Kb), BALB/c (H2Kd), IFN-*γ*−/−, Prf1−/−, and Rag2IL2Rg doubly deficient mice. MyD88KO mice. IL-36R −/− mice (C57BL/6-Il1rl2<tm1Hblu>)	B16 and 4T1 cells primary lymphocyte culture from C57BL/6 mice.	(i) IL-36*γ* effectively promoted IFN-*γ* production by *γδ* T and NK cells. (ii) Tumoral expression of IL-36*γ* greatly inhibited tumor growth and metastasis in vivo, mainly through IFN-*γ*. (iii) IL-36*γ* can boost the efficacy of tumor vaccination

Nieda M. Experimental Dermatology, (2015)	Research	NA	NA	PBMCs from HDs and Pts with metastatic melanoma (stage IV). V*γ*9V*δ*2 (or CD8^+^) T cells stimulated with autologous CD56 high^+^ IFN-DCs or mIL-4DCs in the presence of zoledronate (1 lM) and IL-2 (1000 U/ml) for 10–14 days.	CD56 high^+^ IFN-DCs efficiently promote the expansion of CD56^+^ V*γ*9V*δ*2 T cells in the presence of zoledronate and IL-2.

Conlon K.C. et al, JCO. (2015)	Clinical	n. 11 Patients with metastatic Melanoma were treated with rhIL15 and frequency and cytokines released were analyzed (phase I study).	NA	NA	There was rhIL15-mediated activation of monocytes, NK and *γδ* T cells. No objective remissions in all patients, with best response being stable disease.

Gehrmann U. et al, Cancer Res. (2013)	Research	NA	C57Bl/6, V*α*14-Ca^−^/^−^ and CD1d^−^/^−^ mice. Exosomes loaded with *α*GC + OVA were used to treat the tumor-bearing mice and evaluate tumor growth.		(i) Exosomes loaded with *α*GC and OVA[Exo(*α*GC-OVA)] induce an early iNKT-cell response, dendritic cell, MZB cell activation as well as NK- and *γδ* T-cell activation and proliferation *in vivo* (ii) Exo(*α*GC-OVA) also decrease tumor growth and induce T-cell infiltration in a mouse melanoma model

Lança T. et al, Journal of Immunology. (2013)	Research	NA	C57BL/6 (B6) mice. B6.TCRd^−^/^−^, B6.Ccl2^−^/^−^, B6.Ccr2^−^/^−^ Transplantable B16 melanoma model was used to profile chemokines in tumor lesions and assess their impact on *γδ* TIL recruitment in vivo.		Cytotoxic *γδ* T cells infiltrate B16 lesions and delay tumor growth *in vivo*. B16 lesions in TCRd-deficient mice would accumulate chemokines normally consumed by *γδ* T cells during tumor infiltration, the CCR2 ligands: CCL2 and CCL12 were significantly overexpressed in TCRd-deficient mice. Human V*δ*1 T cells (but not V*δ*2) express CCR2 and migrate toward CCL2 *in vitro*.

Kunzmann V. et al, J Immunother. (2012)	Clinical	n. 21 Patients with metastatic renal cancer, metastatic MM or AML were enrolled to receive zoledronic acid plus IL-2 with 58 treatment cycles being administered.	NA		All patients showed an expansion in circulating *γδ* T cells *in vivo *after zoledronate and low-dose IL-2. Quantitative analysis of cytokine serum levels demonstrated that *in vivo* activation of *γδ* T lymphocytes by zoledronate plus IL-2 induced a significant increase in the proinflammatory cytokine IFN- *γ*. Serum VEGF levels negatively correlated with the clinical benefit.

Nicol AJ et al. British Journal of Cancer. (2011)	Clinical	Phase I trial metastatic cancer patients (n=18) (n=7 melanoma). *γδ* T cells were expanded ex vivo and adoptively transferred in combination with zoledronate administration	NA	NA	(i) Combination therapy with V*γ*9V*δ*2 T cells and zoledronate is well tolerated. (ii) Better results in patients not pretreated with zoledronate. Γ*δ* T cells had an activated effector memory phenotype, expressed chemokine receptors predictive of Vg9Vd2 homing to peripheral tissues and were cytotoxic in vitro against tumour targets, but most patients progressed despite therapy. (iii) the percentage of Tregs in the blood correlated with poor *γδ* T cells expansion

Abbreviations: Melanosomal membrane-protein glycoprotein 100 (gp100), T-cell receptor (TCR), protein melanoma-associated-chondroitin-sulfate-proteoglycan (MCSP), chimeric antigen receptor (CAR), peripheral blood mononuclear cell (PBMC), magnetic-activated cell sorting (MACS), intralesional (IL) *Mycobacterium bovis* bacille Calmette–Guérin (BCG), Zoledronate (ZOL), liposomal ZOL (L-ZOL), liposomal ALD (L-ALD), interleukin (IL), interferon gamma (IFN*γ*), natural killer (NK), healthy donor (HD), patient (Pt), dendritic cells (DC), recombinant human interleukine 15 (rhIL15), invariant NKT (iNKT), alpha-galactosylceramide (aGC), ovalbumin (OVA), exosomes (EXO), C-C motif chemokine ligand (CCL) and receptor type 2 (CCR2), vascular endothelial growth factor (VEGF).
